# The first complete chloroplast genome of the fern genus *Polystichum* (Dryopteridaceae)

**DOI:** 10.1080/23802359.2019.1704198

**Published:** 2020-01-08

**Authors:** Wen-Qin Tu, Yin-Zhang Wang, Yun-Dong Gao, Li-Bing Zhang, Yong-Mei Zhang

**Affiliations:** a Key Laboratory of Mountain Ecological Restoration and Bioresource Utilization, Chengdu Institute of Biology, Chinese Academy of Sciences, Chengdu, PR China;; b Missouri Botanical Garden, St. Louis, MO, USA

**Keywords:** *Polystichum*, Dryopteridaceae, chloroplast, genome

## Abstract

The first chloroplast genome of the fern genus *Polystichum* Roth (Dryopteridaceae) is reported here. *Polystichum deltodon* (Baker) Diels belongs to subgenus *Haplopolystichum* (*Polystichum*; Dryopteridaceae), many species of which are endangered or critically endangered species. The complete chloroplast genome of *P. deltodon* was determined for the first time in this work, which is revealed a circle quadripartite structure of 154,143 bp in length comprising a large single-copy region (LSC) of 86,990 bp, a small single-copy region (SSC) of 21,593 bp and a pair of inverted regions (IRs) of 22,780 bp, respectively. Based on the reported chloroplast genomes of Dryopteridaceae, phylogenetic analyses suggested that *P. deltodon* was located nearly to the genus *Crytomium*, which is in agreement with previous systematic research.

The fern genus *Polystichum* Roth (Dryopteridaceae) comprises approximately 500 species, commonly growing in the temperate regions and subtropical lowlands and montane to alpine areas in the N Hemisphere, mostly in S and SW China, Himalaya, Japan, and Vietnam. Rich diversity of this genus is also found in Central and South America (Zhang and Barrington 2013). It was believed that there were 208 species (139 endemic) in two subgenera for the *Flora of China* (Zhang and Barrington 2013). *Polystichum* subg. *Haplopolystichum* contains about 40 mostly cave species, 26 of which are endangered or critically endangered.

Recent studies revealed the benefits of DNA sequence data for phylogenetic and taxonomic studies of *Polystichum* and its relatives (Le Péchon, He, et al. 2016; Le Péchon, Zhang, et al. 2016). However, plastomes of this probably the third largest fern genus are still missing. Herein, we generated the chloroplast genome of *P. deltodon* (Baker) Diels (Diels 1900) using Illumina HiSeq platform, which can be essential to the identification, phylogeny, and evolution of *Polystichum*.

Fresh leaves of *P. deltodon* were collected from Bifengxia, Bifeng Village, Bifengxia Town, Yucheng Distr., Ya’an Prefecture, Sichuan, China (102°59′8″E, 30°4′39″N, elev. 1050 m) on 28 June 2019, in acidic soil derived from sandstone substrate. The voucher specimen (acc. # ZYM002) and DNA samples were deposited at the herbarium of Chengdu Institute of Biology, CAS (CDBI). Total genomic DNA was extracted by Plant Genomic DNA Kit (TianGen, Beijing, China). Sequencing libraries were generated using NEBNext® Ultra™ DNA Library Prep Kit for Illumina (NEB, Ipswitch, MA). The library preparations were sequenced on an Illumina HiSeq platform, and paired-end reads (PE150) were generated (Novogene, Beijing, China). The chloroplast genome of *P. deltodon* was mapped and reconstructed using Geneious Prime 2019.2.3 (Kearse et al. 2012) and annotated with *Cyrtomium fortunei* (GenBank: NC_037510.1) as the reference. The complete chloroplast genome of *P. deltodon* is deposited in GenBank with acc. no. MN640792, which was 154,143 bp long in a circular form, consisted of four distinct regions: the large single-copy region (LSC) of 86,990 bp, the small single-copy region (SSC) of 21,593 bp, and two copies of inverted regions (IRs) of 22,780 bp, respectively. The total GC content of *P. deltodon* was 42.5%, with 41.8% for the LSC, 39.6% for the SSC, and 45.1% for each IR. The genome contained 8 rRNAs, 38 tRNAs, and 77 protein-coding genes, accounting for a total of 123 genes. The tRNA coding genes were mainly distributed in the LSC (including 57), 5 in IRs, and 15 in the SSC; while the rRNA coding genes were only located in the IRs. The phylogenetic analysis was conducted with *P. deltodon* and the related species in Dryopteridaceae included. The complete chloroplast genome sequences of 11 species from five genera were aligned using MAFFT (Katoh et al. 2019). A neighbor-joining (NJ) tree was performed using MEGA X (Kumar et al. 2018) with 500 bootstrap replicates, and the support values are shown next to the branches in Figure 1. The result suggests that *P. deltodon* is closely related with *Cyrtomium*, which is well in agreement with previous studies based on morphological and molecular evidence (Le Péchon, He, et al. 2016; Le Péchon, Zhang, et al. 2016).

**Figure 1.**
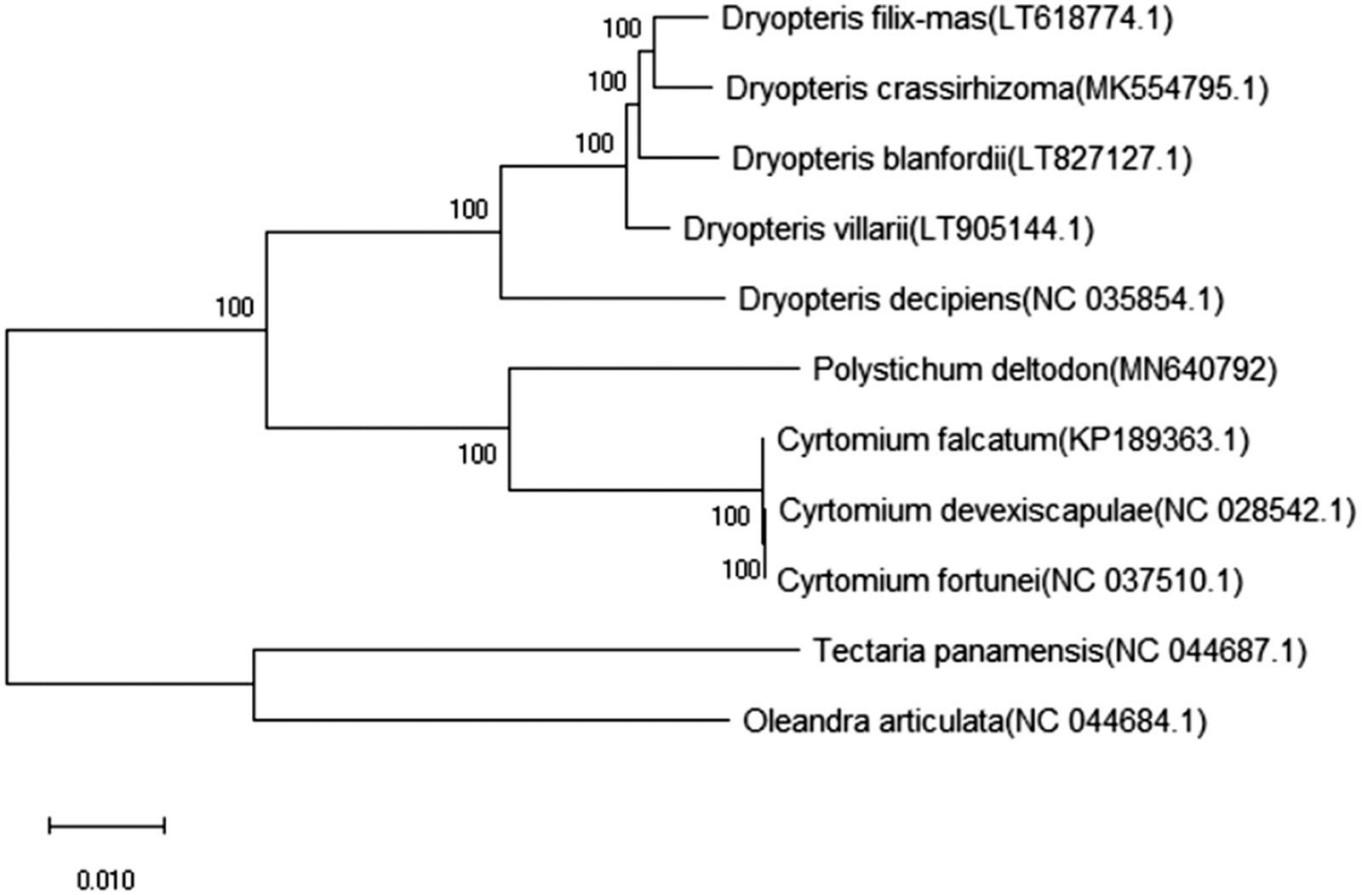
Neighbor-joining tree of *Polystichum deltodon* and related species based on complete chloroplast genome sequences. Numbers on the nodes show the bootstrap values from 500 replicates.
